# Understanding pre-hospital blood transfusion decision-making for injured patients: an interview study

**DOI:** 10.1136/emermed-2023-213086

**Published:** 2023-09-13

**Authors:** Max E R Marsden, Suzie Kellett, Rahul Bagga, Jared M Wohlgemut, Richard L Lyon, Zane B Perkins, Katie Gillies, Nigel RM Tai

**Affiliations:** 1 Centre for Trauma Sciences, Blizard Institute, Queen Mary University of London, London, UK; 2 Academic Department of Military Surgery and Trauma, RCI, Defence Medical Services, Birmingham, UK; 3 Department of Anaesthesia, Univeristy Hospital Southampton, Southampton, UK; 4 Department of Orthopaedics, University Hospitals Birmingham NHS Foundation Trust, Birmingham, UK; 5 Air Ambulance Kent, Surrey and Sussex, Redhill, Surrey, UK; 6 School of Health Sciences, University of Surrey, Guildford, UK; 7 London's Air Ambulance, Barts Health NHS Trust, London, UK; 8 Health Services Research Unit, University of Aberdeen, Aberdeen, UK

**Keywords:** major trauma management, resuscitation, pre-hospital, diagnosis

## Abstract

**Background:**

Blood transfusion for bleeding trauma patients is a promising pre-hospital intervention with potential to improve outcomes. However, it is not yet clear which patients may benefit from pre-hospital transfusions. The aim of this study was to enhance our understanding of how experienced pre-hospital clinicians make decisions regarding patient blood loss and the need for transfusion, and explore the factors that influence clinical decision-making.

**Methods:**

Pre-hospital physicians, from two air ambulance sites in the south of England, were interviewed between December 2018 and January 2019. Participants were involved in teaching or publishing on the management of bleeding trauma patients and had at least 5 years of continuous and contemporary practice at consultant level. Interviews were semi-structured and explored how decisions were made and what made decisions difficult. A qualitative description approach was used with inductive thematic analysis to identify themes and subthemes related to blood transfusion decision-making in trauma.

**Results:**

Ten pre-hospital physicians were interviewed and three themes were identified: *recognition-primed analysis*, *uncertainty* and *imperfect decision analysis*. The first theme describes how participants make decisions using selected cues, incorporating their experience and are influenced by external rules and group expectations. What made decisions difficult for the participants was encapsulated in the uncertainty theme. Uncertainty emerged regarding the patient’s true underlying physiological state and the treatment effect of blood transfusion. The last theme focuses on the issues with decision-making itself. Participants demonstrated lapses in decision awareness, often incomplete decision evaluation and described challenges to effective learning due to incomplete patient outcome information.

**Conclusion:**

Pre-hospital clinicians make decisions about bleeding and transfusion which are recognition-primed and incorporate significant uncertainty. Decisions are influenced by experience and are subject to bias. Improved understanding of the decision-making processes provides a theoretical perspective of how decisions might be supported in the future.

**WHAT IS ALREADY KNOWN ON THIS TOPIC**

- Large trials of the effectiveness of pre-hospital blood transfusion have demonstrated variable effectiveness.
- Little is known about how pre-hospital clinicians decide which patients to transfuse.

**WHAT THIS STUDY ADDS**

- This qualitative study involving interviews from 10 experienced pre-hospital physicians contributes specific insights into the factors that influence decision-making regarding pre-hospital blood transfusion.
- The study highlights the complexities of decision-making under conditions of uncertainty, with transfusion decisions influenced by clinician experience, clinical guidelines, group expectations and biases.

**HOW THIS STUDY MIGHT AFFECT RESEARCH, PRACTICE OR POLICY**

- Enhancing our understanding of pre-hospital decision-making helps us develop strategies for better decision-making in the future.

## Introduction

Decision-making during resuscitation presents formidable challenges, particularly in the demanding context of pre-hospital trauma care. The high-stress, noisy and time-pressured conditions are known to impact decision-making ability, which is compounded by limited access to diagnostic adjuncts and immediate peer support.^1^

A critical aspect of pre-hospital trauma care is the early identification and management of life-threatening bleeding.^2^ This includes the decision to initiate pre-hospital blood transfusions.^3^

However, deciding whether to initiate transfusion is particularly difficult.^4^ Diagnostic uncertainty,^5^ rapid changes in the patient’s condition and the potential consequences of an incorrect decision are factors that influence the appropriate use of this limited resource. Inaccurate diagnoses can lead to unnecessary transfusions, while underestimating the need for blood may result in inadequate resuscitation. Moreover, errors made in the pre-hospital setting can propagate into the hospital, leading to further inappropriate treatment until the patient’s true condition is recognised.^6^

Understanding how pre-hospital clinicians arrive at decisions is crucial for optimising clinical performance. Traditional decision-making theories suggest rational comparison of multiple options to select the most effective one. More recent theories have highlighted the role of pattern recognition and mental shortcuts in decision-making, and how these cognitive processes may occasionally be prone to error and biases.^7^ Naturalistic decision-making (NDM) emphasises the role of tacit knowledge acquired through experience. NDM researchers have demonstrated that experts in various fields demonstrate better performance in real-world scenarios compared with what traditional theories predict.^8^ NDM focuses on understanding decision requirements and developing technology to support decision-making processes.

Improving outcomes for bleeding trauma patients requires appropriate decision-making from the earliest phase of care. By recognising the weaknesses in pre-hospital clinical decision-making and understanding the factors that influence decisions, we can inform strategies to enhance accuracy.^9^ This study aimed to gain insights into how expert pre-hospital physicians make decisions regarding bleeding severity and transfusion requirements, and the factors that influence these decisions.

## Methods

### Study conduct

The study follows the Standards for Reporting Qualitative Research guideline.^10^

### Qualitative approach

Multiple methodological frameworks exist within qualitative research. In this study, we have taken a qualitative description approach^11^ to provide detailed and interpreted insights into pre-hospital decision-making.^12^ The qualitative description approach is free from any one theoretical framework, and focuses the analysis on the description given by the participants. The research ‘strives to stay close to the surface of the data’ by using a combination of literal descriptions, coupled with interpretation of the participants’ ascribed meaning.^13^ In an applied health services research context, qualitative description provides a method of addressing the specific a priori research questions while also allowing for de novo data to be incorporated. It is acknowledged that the researcher characteristics (online supplemental table S1) influence the research findings in such study designs.

10.1136/emermed-2023-213086.supp1 Supplementary data

### Study setting

The study was conducted at two air ambulance sites referred to as site A and site B. Both organisations are in the South of England and provide a continuous physician and paramedic response to critically injured patients. Site A provides care within an urban major trauma network whereas site B serves a larger geographic area with a lower population density and greater distances to major trauma centres. Both services have well-established pre-hospital blood transfusion capabilities. The decision to give blood is solely made by physicians at both sites.

### Data collection

The study used a purposive sampling strategy.^13^ Currently practising pre-hospital consultants with at least 5 years continuous pre-hospital experience were approached by email. Consultants were selected based on their involvement in teaching or publishing on the management of bleeding trauma patients. No invitations were declined. Participants were given a study number to maintain their anonymity.

Semi-structured interviews were conducted by MERM between December 2018 and January 2019 (online supplemental table S2).^14^ The interview questions were generated by discussion between the authors. The questions addressed how experienced pre-hospital trauma doctors make decisions about bleeding and transfusion and explored their perspective on the factors that make such decision-making difficult or challenging.

10.1136/emermed-2023-213086.supp2 Supplementary data

Interviews were conducted in person at the air ambulance headquarters by the primary researcher and audio-recorded. The recordings were transcribed verbatim (MERM and RB) and imported to NVivo V.12 for Mac (QSR International, Doncaster, Australia) to facilitate data analysis.

### Data processing and analysis

Inductive thematic analysis was approached using the six phases of thematic analysis.^15^ Phases I and II involved data familiarisation and inductive generation of initial codes within the interview transcripts. Initial open data coding was performed independently by three coders (MERM, RB and SK) followed iteratively by focused coding and comparison to refine ideas and develop themes. Phases III and IV sought to search for and then refine themes. A subsequent round of coding took place where refinement of codes was achieved using a common codebook between coders. Differences of opinion were settled by discussion between coders. In phase V, the themes were defined and named and the study was written up in phase VI. Transcribed interviews were returned to the participant with the coded text annotated and the participant asked to validate the accuracy of transcription. A frequency analysis was performed to quantify codes and themes generated from the data.^15 16^

### Researchers’ characteristics and study context

MERM is a British man training in General Surgery and Major Trauma in London, UK. MERM has basic pre-hospital emergency experience. MERM is influenced by the works of Kahneman (Heuristics and Biases) and Klein (NDM) in the critical analysis of decision-making. MERM has undergone training in qualitative research approaches during his doctoral research (online supplemental table S1). The study was conducted as part of a doctoral research degree, assessing the impact of decision support tools on pre-hospital trauma patients at the Centre for Trauma Sciences, Blizard Institute, Queen Mary, University of London.

### Patient and public involvement

Patients and/or the public were not involved in the design, or conduct, or reporting, or dissemination plans of this research.

## Results

### Sample characteristics

A total of 10 interviews were undertaken: 6 participants from site A and 4 from site B. The study included nine male participants, which reflects the higher proportion of male pre-hospital clinicians at both sites. Two participants were anaesthetists with the other participants trained in emergency medicine. The duration of pre-hospital practice for site A and site B was a median of 16 years and 9 years (table 1). Interviews lasted a median of 30 (IQR 28–35) min. Once eight interviews had been conducted, further data collection provided diminishing returns. The final two interviews did not provide additional themes or subthemes.

**Table 1:** Participant demographics

Characteristic	Site A	Site B
Participants, n	6	4
Gender (M:F)	5:1	4:0
Base specialty		
Emergency medicine	5	3
Anaesthesia	1	1
Years of pre-hospital experience median (IQR), years	16 (10–18)	9 (8–10)

F, female; M, male.

### Overall findings

**Figure 1:**
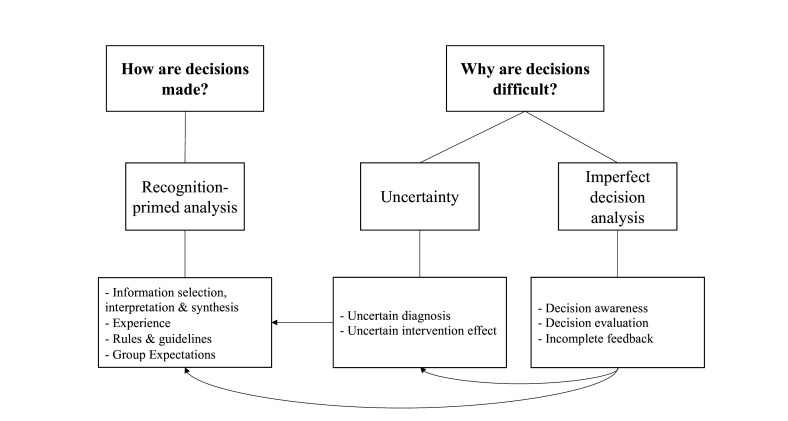
Connections between themes identified by thematic analysis. The top row of boxes denotes the study questions, the middle row the overarching themes and the bottom row the subthemes. Arrows demonstrate the interconnectivity between themes.

To address how expert clinicians make decisions about bleeding and transfusion, the theme *recognition-primed analysis* was identified with the subthemes ‘information selection, interpretation and synthesis’, ‘experience’, ‘rules and guidelines’ and ‘group expectations’. The second research question, ‘what makes decisions about bleeding and transfusion difficult for expert clinicians?’ generated two themes: *uncertainty,* with the subthemes ‘uncertain diagnosis’ and ‘uncertain intervention effect’, and *imperfect decision analysis* with three subthemes ‘decision awareness’, ‘incomplete feedback’ and ‘decision evaluation’ (figure 1). The relationship between the data codes and the main themes as well as the frequency of codes is provided in tables 2–4. Themes are presented below according to the research questions.

**Table 2:** Codes relating to recognition-primed analysis theme

Subtheme	Code	Interviews (n)	Frequency (n)
	**Information selection (cues**)		
Information selection, interpretation and synthesis	Mechanism of injury	10	20
	Physical signs of injury (including hateful eight)	10	40
	Visual and non-quantitative signs of bleeding	9	43
	Using trends and anticipating trajectory	8	18
	Autonomic response to injury	5	9
	Quantitative signs and diagnostic devices suggestive of bleeding	3	6
	**Information interpretation**		
	Develop a differential diagnosis	8	24
	External factors such as time and distance	6	16
	Primacy of the primary survey	6	14
	Individual patients respond differently	6	10
	Assessment of critical hypoperfusion	5	9
	**Information synthesis**		
	Combining information to make a decision	9	51
	Expectation prior to patient assessment	5	9
Experience	Influence of experience	9	34
	Interpretation of signs changes with experience	6	9
	Clinical culture	2	5
Rules, guidelines and evidence	Rule-based decision-making	7	16
	Example of following an SOP or protocol	5	12
Group expectations	Post hoc scrutiny	3	4

‘Interviews’ refers to the number of interviews in which a code was identified. ‘Frequency’ refers to a count of each code in every interview.

SOP, standard operating procedure.

**Table 3:** Codes relating to uncertainty theme

Subtheme	Code	Interviews (n)	Frequency (n)
Uncertain diagnosis	Few pre-hospital diagnostic aids for major bleeding	10	31
	Haemorrhage mimics can confuse diagnosis	8	22
	Difficult to prognosticate	8	19
	Need to compile information and make a global assessment	8	9
	Differentiating minor bleeding from major bleeding	7	13
	Haemodynamic assessment maybe misleading	6	17
	Fallibility of haemodynamic parameters	6	16
	Clinical examination is not 100% accurate	6	7
	Individual patient variability	5	15
	Clinical unknowns relating to the patient (eg, extent of their injury)	4	12
Uncertain intervention effect	**Immediate effect of a given intervention**		
	Benefit of transfusion (includes clotting, oxygen delivery, perfusion, preload)	9	52
	Weighing up benefit and harm	9	16
	Risks of transfusion (includes dilution, increased BP, immunological effects, BBV, VTE, metabolic)	5	17
	Recognition of threshold variation between clinicians when to start transfusion	5	7
	**Future effect of a given intervention**		
	Transfusion mitigates future pathological states (eg, TIC/MODS)	6	8
	Blood transfusion improves patient’s physiological state in moderate bleeding patients	3	3
	**No perceived effect of a given intervention**		
	Rapid transport to hospital is key	4	6

‘Interviews’ refers to the number of interviews in which a code was identified. ‘Frequency’ refers to a count of each code in every interview.

BBV, bloodborne virus; MODS, multiple organ dysfunction syndrome; TIC, trauma-induced coagulopathy; VTE, venous thromboembolism.

**Table 4:** Codes relating to imperfect decision analysis theme

Subtheme	Code	Interviews (n)	Frequency (n)
Decision awareness	Automatic decision-making	7	20
	Evidence of framing bias	5	6
	Evidence of substitution bias	2	3
Decision evaluation	Suggestion of flawed decision evaluation	3	5
Incomplete feedback	The decision is judged on clinical outcome	2	2

‘Interviews’ refers to the number of interviews in which a code was identified. ‘Frequency’ refers to a count of each code in every interview.

### **Question 1: How do expert clinicians make decisions about bleeding and transfusion?**

### Theme 1: recognition-primed analysis

Participants make recognition-primed decisions using selected cues, incorporating their experience, and are variably influenced by external rules and group expectations.

#### Subtheme 1: information selection, interpretation and synthesis

Participants provided detailed descriptions of the overt analytical processes they used to establish a diagnosis of major bleeding (table 2). In making a diagnosis of major bleeding, participants talked about information (cues) being gathered first and then analysed before decisions are made. Participants reported selecting cues from multiple sources and actively considering familiar patterns of both the specific clinical diagnostic indicators and the broader clinical context. This process of pattern recognition often started early in the pre-hospital mission cycle from the initial sparse information given to the pre-hospital clinical team.

I mean part of the assessment probably starts before you even get to the scene … knowing what job you’re going to … knowing your mechanism or what the alleged mechanism is that starts you thinking. (Participant 8, site B)

Once participants arrived at the patient’s location, they described using multiple cues to update their perception of the risk of major bleeding and create a more complete mental model. All participants highlighted that some indicators were more predictive of life-threatening haemorrhage than others.

A lot of the surrogates that we use to identify bleeding … are not particularly sensitive and so you need to add as many layers to the picture as possible, it’s almost like pieces of a puzzle that allows you to then stand back and look at the whole picture once you’ve got each piece. (Participant 6, site A)

Participants at one of the sites reported that their institution encourages clinicians to recognise a set of eight clinical diagnostic indicators that are believed to be predictive of life-threatening haemorrhage (online supplemental table S3). Participants also acknowledged that not all the cue selection, interpretation and synthesis is a conscious process.

10.1136/emermed-2023-213086.supp3 Supplementary data

I’d like to say that I’m always aware of the colour of [the patient’s] skin, and those sorts of things, but I’m probably not. Though sometimes it is obvious that someone looks very pale and you pick up those cues, but it’s not always the case. (Participant 1, site A)

The two sites reported differences in their use of point-of-care testing and the value these tests contributed to the overall clinical assessments. Participants at site B used point-of-care lactate measurement and ultrasound imaging to influence their decision-making. Participants at site A did not use these tools and expressed uncertainty about their value.

I really like the lactate, because I think it just gives me that extra dimension for those patients where I’m either on the fence or the patient doesn’t, to my external assessment, declare themselves one way or the other. (Participant 3, site B)

We don’t currently do … blood gas testing but … I’ve always tried to think … if I could take the gas now what [would] the values be? … I hope that I transfuse patients who have a big base deficit or high lactate. … until the machines get a bit better I'm not sure they’re going to add a lot to our clinical armamentarium. (Participant 5, site A)

#### Subtheme 2: experience

Participants described a rapid intuitive decision-making process that was linked to having the benefit of experience. Participants reported that with more experience they had changed what cues they sought to inform their decision-making and the way clinical information was interpreted and synthesised.

…previously I would have been more swayed by physiology and perhaps less so by the findings on the primary survey and perhaps increasingly, I’ve moved slightly in the opposite direction. (Participant 2, site B)

As clinicians gained experience, the decision about when to start a blood transfusion also changed. One participant believed that the experience gained in their subspecialty (emergency medicine or anaesthesia) may also be responsible for the reported differences in opinion about when to start a blood transfusion.

I think I have less of less of an existential angst about giving blood than some of my colleagues and I think everyone from their background specialty has got different context. (Participant 6, site A)

To this individual, the differences appeared in part to originate from divergent anticipation of the patient’s in-hospital therapy.

So I look at the overall trajectory of the patient … they didn’t give any blood products to the patient pre-hospital and what happens is the first blood pressure comes up [in the ED] at 68 systolic and all that happens is that you connect the Belmont and put four units of blood straight into the patient. And once that patient gets to my operating theatre, the surgeon unzips the patient and blood is hosing out. … I have no hesitation in giving blood for those sorts of patients. (Participant 6, site A)

#### Subtheme 3: rules, guidelines and evidence

Decisions about when to initiate a blood transfusion were described less precisely than descriptions of how to recognise bleeding.

She was awake and talking, she was never hypotensive, so I wouldn’t give her blood … if I think they’re bleeding and I think they are going to benefit from a blood transfusion I am saying … I think they’re going to benefit from enhanced organ perfusion, if their blood pressure is higher than it currently is now. (Participant 5, site A)

Standard operating procedures (SOPs) are regularly used to reduce unwanted variability in medical provision. For pre-hospital blood transfusion, SOPs include criteria on when to start a transfusion. While discussing triggers for blood transfusion, most participants referred to their SOP but suggested that they were not rigidly adhered to (table 2). Alongside the institutional SOPs, several participants described loose self-generated rules that influenced their decisions.

If you’ve got a blood pressure that looks within a reasonable range (and essentially that is something in the 100 plus range) then it just lowers your clinical suspicion that this person has bled to a point that they’re going to need blood products. It’s not an absolute rule. (Participant 2, site B)

The limitations of detailed clinical trial evidence were frequently mentioned. Participants described that the lack of applicable high-quality evidence made it difficult to apply evidence-based decision-making to a specific patient.

We just still don’t really know whether giving plasma is going to help them, whether packed cells are going to help… we know that these patients will be okay for a period of time, quite how long that is… I’m not sure anyone knows … there’s good evidence for [permissive hypotension] for penetrating trauma, we know that… it’s more difficult for blunt trauma. (Participant 4, site B)

#### Subtheme 4: group expectations

Some of the participants described how their decision-making was influenced by pressure they felt from others in their clinical environment. The anticipation of post hoc scrutiny of the patient’s management during formal retrospective reviews was raised by two participants. For some participants, there appeared to be a tension between what they thought was best practice and what they believed their peers would construe as best practice. Faced with this tension, and regardless of seniority, some participants appeared to make decisions to fit in with the wider group’s expectations while others were content to do what they thought was right.

it seems to be almost like a badge of honour for bringing someone in and having avoided giving them the blood. But actually their physiology is deranged, and they need the volume replacement and I would rather replace them with blood and blood products. (Participant 6, site A)

### **Question 2: What makes decisions about bleeding and transfusion difficult for expert clinicians?**

### Theme 2: uncertainty

When making decisions about transfusions participants handle significant uncertainty both relating to the patient’s true underlying physiological state and the treatment effect of blood transfusion.

#### Subtheme 1: uncertain diagnosis

There was universal agreement among participants that uncertainty was a significant barrier to decision-making (table 3). Uncertainty was divided into two subthemes: uncertainty surrounding the patient’s diagnosis and uncertainty relating to the potential benefit of administering a transfusion. This combined uncertainty precluded effective prognostication, which in turn directly impacted on several key pre-hospital decisions. Participants described situations in which they did not have enough reliable information to make a confident decision.

it would be lovely to remove some of the complicating factors … I’d love to know how well the [patient’s] tissues are being perfused. (Participant 9, site A)

Often uncertainty resulted from the imprecision of physiological observations and missing critical injury information. As a result, participants described difficulty accurately portraying the patient’s true state.

If you’re to avoid an exsanguination mimic, you need to establish that they’ve had a mechanism of injury consistent with some injuries and you’ve found those injuries. Because you could have all of that [abnormal] physiology … and not have any injuries… that’s where it goes wrong; people just look at the physiology. (Participant 10, site A)

Patients injured with high energy blunt force were highlighted as particularly difficult to diagnose, as they often have multiple injuries and may lack obvious cues such as external haemorrhage. This lead to uncertainty of the cause of their abnormal physiological observations.

blunt trauma: that’s the difficult group and occasionally they declare themselves for you by becoming more haemodynamically unstable and they have a hypotensive episode, become increasingly tachycardiac, or their end tidal drops. And that can be a trigger to starting blood. (Participant 2, site B)

#### Subtheme 2: uncertain intervention effect

Participants differed in their rationale for giving pre-hospital blood. Some examples of the proposed benefit were to repay the oxygen debt of shock, stabilise coagulopathy or to address immediate or subsequent organ failure.

I’m not sure anybody knows this as fact, but there’s this worry that by having that period of having under resuscitated [a patient], you set in chain a load of things that are going to cause them difficulty in the future; be that coagulopathy or multi-organ dysfunction. (Participant 2, site B)

There was universal agreement that transfusion should be started immediately in patients with such severe blood loss that circulatory arrest was impending or had occurred. However, outside of severe blood loss, participants did not clearly articulate or agree when blood products should be given.

My threshold [to transfuse] is lower [than my colleagues] because I want them to arrive in a physiological state that is better than it is now. (Participant 7, site A)

If I think you’re shocked for another reason [than hypovolaemia] or you are shocked and I’ve stopped the bleeding and you’re not really in an awful state, I’m not going to give you pre-hospital blood. And I think that’s because I’m trying to do more good than harm overall. (Participant 10, site A)

As a result of the uncertainty of the benefit of blood transfusion, there was a variable reliance on either gestalt or reversion to SOPs to decide. Asked to reflect on a scenario in which the benefit of a blood transfusion was uncertain, participants agreed that the risks of undertransfusion were greater than the harms of an unnecessary transfusion.

there are some of these mimics with head injuries and so on that you’re not going to tolerate someone sitting there with a systolic blood pressure that’s not recording, looking awful with blood next to you and not give it to them. I think you’ve got to be pretty brave to do that. (Participant 6, site A)

### Theme 3: imperfect decision analysis

For participants the process of decision-making is intermittently unconscious, imperfectly evaluated and challenged by incomplete learning loops.

#### Subtheme 1: lapses in decision awareness

Perceived shortcomings in their own clinical abilities were discussed openly but participants rarely discussed the process of decision-making (table 4). Half of the participants referred to their decision-making as reliant on unconscious processes.

A lot of the assessment actually happens in the first few seconds … experienced clinicians will make a judgment very very quickly … then you try to reinforce that with your full formal assessment of the patient, probably try to work out whether your gut reaction was the right one. (Participant 10, site A)

Participants were aware that decisions made unconsciously were prone to error.

… I think it’s gut feel and because of that it’s not always particularly sensitive and sometimes we get it wrong. (Participant 8, site B)

Associated with lapses of awareness of making a decision, participants often struggled to articulate the factors which affected their decision-making. Where decisions were explained, there were examples of seemingly limited awareness of biases affecting decisions.

I mean part of the assessment, really in your head, probably starts before you even get to scene because you, kind of, know what job you’re going to. (Participant 8, site B)

In the case of uncertainty… I’m going to give it [blood] to them… and I think that’s because of our patient group. Most the time, the chance of them bleeding is higher than the chance of just having impact brain apnoea or an alternative, and I think also that we’re quite good at excluding other causes. (Participant 5, site A)

Often such statements, which hint at a blind spot to the potential for bias, did not seem apparent to the participant. However, sometimes the risk of bias was explicitly stated:

Because we carry blood and because you’ve got a solution. You can make the patient fit your solution. But that patient may not be bleeding. (Participant 8, site B)

#### Subtheme 2: incomplete decision evaluation

Participants provided multiple examples where they showed that they had reflected on a decision after it had occurred. However, none of the participants mentioned using a standardised method to evaluate the quality of their decisions more thoroughly.

The pendulum swings based on your previous experiences. You can have a time where you feel that maybe blood was started by you or a colleague sooner than it was needed and then other times you feel it was started late. And that can impact on your next decision-making. (Participant 2, site B)

Similarly, the participants did not mention the use of a decision-making framework, which could have provided a standardised approach to making decisions.

#### Subtheme 3: incomplete feedback challenges learning

Participants recognised that their learning is often hampered by incomplete feedback on their decision-making.

Are we actually getting it right or not? I’d love to know. Sort of almost retrospectively so you’re gonna learn from these cases. (Participant 10, site A)

## Discussion

In this study, participants identified a substantial challenge to pre-hospital decision-making arises from clinical uncertainty. That uncertainty relates to both confidently identifying a patient’s true underlying physiological state and the treatment effect of blood in patients not at immediate risk of hypovolaemic cardiac arrest. To cope with this uncertainty, the decision-making process employed by our participants can be characterised as a recognition-primed approach.^8^ This means that they rely on identifying cues which they exploit to weigh their judgements. By doing so, they minimise the time spent processing a large volume of information, much of which is ambiguous and lacks specificity.

Participants agreed that multiple factors can impinge on their decision-making processes, of which exposure to similar clinical situations (experience) matters significantly. Klein demonstrated that fire fighters faced with challenging scenarios used recognition-primed strategies 80%–90% of the time.^8^ This observation has recently been replicated in healthcare. In a study of anaesthetists faced with difficult airway management situations, 91% of the decisions were recognition primed.^17^ Anaesthetists characteristically made a direct link between familiar cues and action generation. The first action the anaesthetists considered was usually the action they implemented.^18^

Our interviews revealed the uniquely challenging clinical environment pre-hospital clinicians face. In this context, an experiential-based, pattern-recognition approach to decision-making is fraught with challenges. Pattern-matching relies on building up an unbiased understanding of the multiple interacting variables and the intricate non-linear relationships between these variables and a clinical outcome.^19^ Patient outcomes may not always be obvious to pre-hospital practitioners. Determining the outcomes of patients who receive care in different healthcare settings poses significant challenges. As a result, practitioners may struggle to assess the reliability of their own judgements since they cannot directly trace the relationship between the cues they rely on and the subsequent clinical outcomes. When learning loops are incomplete, clinicians are deprived of the ability to evaluate their own performance and future decisions remain difficult as effective learning does not happen.^20^

The study’s final theme of imperfect decision analysis addresses the challenge of assessing decision quality. Within the interviews participants gave examples of decision-making susceptible to biases of anchoring (overdependence on initial information as a reference point), availability (relying on information that comes to mind readily), framing (when information is presented with certain connotations) and substitution (when difficult judgement tasks are replaced with easier ones). To evaluate decisions effectively, we need to shift from appraising the decision solely based on clinical outcomes to considering the information available at the time of the decision.^21^ Using a decision analysis framework allows for a comprehensive evaluation of a decision that acknowledges and identifies influencing biases, regardless of the outcome.^22^ Effective retrospective case review can be achieved with a framework that promotes narrative reconstruction of events and decision points. Such a technique can improve the quality of future decisions by methodically identifying sources of risk and error.^23 24^

One approach to reduce a clinician’s uncertainty is to employ validated decision support tools, to generate meaningful predictions concerning likelihood of bleeding and likely requirement for transfusion. Use of decision support tools, based on published evidence but powered by individual patient variables, may aid the practitioner’s need to correctly interpret the clinical situation at hand. Improving clinical situational awareness may improve the selection of appropriate treatment goals and increase the likelihood that the chosen management course is the most fitting for the patient in front of them.^6^

### Limitations

The study’s sample is relatively small by quantitative analysis standards. For this qualitative study, the sample size is sufficient using the concept of information power: the more information the sample holds, the fewer participants are required. Thus, as the participant sample was highly specific the study had sufficient power.^25^ This purposive sampling strategy does impact the generalisability of the study’s findings. Clinicians working in pre-hospital systems which either do not use pre-hospital blood or have paramedics that start transfusions may have differing views to the participants in this study. Additionally, the sample has a preponderance of men. While this sample represents the sites’ total consultant population, it does raise the broader question of under-representation of women in pre-hospital emergency medicine. The effect of gender on decision-making cannot be explored in this study. Finally, in common with much qualitative research the interview and analysis technique are likely to influence the results. To mitigate the influence on the authors’ beliefs on the results, we aimed to report and interpret the participant’s views in a balanced and transparent fashion (online supplemental table S1).

This study is the first to explore the specifics of pre-hospital decision-making in bleeding trauma patients. Our finding that pre-hospital decision-making can be represented by the recognition-primed decision model permits the utilisation of more targeted interview methodologies suited to this model, such as the critical decision analysis method.^26^ Adopting this interview approach in future may provide a more nuanced understanding of contextual decision-making influences for time-critical emergency conditions where accurate choice of therapy is critical to patient outcome.

## Conclusion

Pre-hospital decision-making regarding bleeding and transfusion is often complicated by uncertainty. Transfusion decisions are influenced by clinician experience, clinical guidelines, group expectations and bias. Future research should focus on exploring the potential of decision support tools to reduce uncertainty and improve clinicians’ ability to correctly interpret the clinical situation.

## Data Availability

Data are available on reasonable request.
