# Supplementary material for: Understanding pre-hospital blood transfusion decision-making for injured patients: an interview study
Source: Emerg Med J. 2023 Sep 13;40(11):777–84. doi: 10.1136/emermed-2023-213086 (PMC10646861; doi:10.1136/emermed-2023-213086)
Supplement: Supplementary data [file emermed-2023-213086supp001.pdf]

Table S1: Researcher characteristics

	Characteristics
General Description	In qualitative studies, the characteristics of the researcher, influence the research findings. *Reflexivity* is the term given to this circular relationship between cause and effect. In this study, a stance of "empathic neutrality" was adopted. Empathic neutrality recognises that there is value-mediated by the researcher on the findings of the study. The empathic neutrality approach aims to make assumptions explicit. To make assumptions clear, the researcher's characteristics, education and relationships with the participants are described.
First author	I am a white British male. I attended Medical School in London and am now a General Surgery Registrar training in London, UK. I have basic pre-hospital emergency experience in a military context and no subspecialty pre-hospital training in a civilian context. I conducted this study as part of a doctoral research degree at the Centre for Trauma Sciences, Blizard Institute, Queen Mary, University of London. I am employed by the UK Ministry of Defence. There are no other sources of funding for this study. All the participants in the study are known to me on a professional basis.
Additional authors	Suzie Kellet provided critical analysis of decision making (anaesthetist, female, unconnected with either study site).<br>Rahul Bagga and Jared Wohlgemut assisted in data analysis and writing (both academic trainee surgeons, male)<br>Pre-hospital domain expertise was gathered from Richard Lyon and Zane Perkins (both pre-hospital physicians, academics, and male)<br>Katie Gillies is a specialist in using mixed methods approaches to study behavioural science applied to trials methodology. KG provided advice on the qualitative approach (academic, female, unconnected with either site).<br>Nigel Tai oversaw the research (academic, PhD supervisor, trauma surgeon, male).<br>Early in the study planning phase Professor Julia Williams provided advice in the qualitative description approach (academic, paramedic academic, female).
