# Supplementary material for: Understanding pre-hospital blood transfusion decision-making for injured patients: an interview study
Source: Emerg Med J. 2023 Sep 13;40(11):777–84. doi: 10.1136/emermed-2023-213086 (PMC10646861; doi:10.1136/emermed-2023-213086)
Supplement: Supplementary data [file emermed-2023-213086supp002.pdf]

Table S2: Interview guide questions

	**Questions**
Clinician Demographics	What is your base speciality?
	How many years' experience do you have as a pre-hospital consultant?
Introduction	I would like you to consider your approach to how you asses, and decide treatments for, trauma patients you see in your pre-hospital clinical role. I'm going to ask you about bleeding. Would that be alright?
Interview Questions	Is it a fair assumption to suggest you assess a trauma patient for bleeding?
	When do you start thinking about whether or not a patient might be bleeding?
	What do you think are the most important clinical pieces of information you use to assess a patient's severity of bleeding?
	How does hypovolaemic shock change your management for a patient?
	What is your intention when giving blood?
	Can we discuss damage control resuscitation?
	Do you think you practice damage-control resuscitation (DCR) pre-hospital?
	When do you think DCR becomes necessary pre-hospital?
	What are the components of your Pre-hospital DCR?
	What influences your decision to transfuse pre-hospital blood?
	What is difficult about deciding if a patient needs a transfusion?
	What do you consider a greater risk to a patient: under transfusion of a bleeding patient or unnecessary transfusion to a patient without significant blood loss?
