# Supplementary material for: Understanding pre-hospital blood transfusion decision-making for injured patients: an interview study
Source: Emerg Med J. 2023 Sep 13;40(11):777–84. doi: 10.1136/emermed-2023-213086 (PMC10646861; doi:10.1136/emermed-2023-213086)
Supplement: Supplementary data [file emermed-2023-213086supp003.pdf]

Table S3: **The hateful eight; factors suggestive of major haemorrhage**

Factor suggestive of major bleeding	Type of Factor	Source
Pale	Qualitative	Visual inspection
Clammy	Qualitative	Visual inspection / palpation
Apparent air-hunger (change in respiratory pattern)	Qualitative	Visual inspection
Venous collapse	Qualitative	Visual inspection
Hypotension (low volume or absent peripheral pulses)	Qualitative	Palpation
Low or falling end tidal CO_2	Quantitative / trend	Electronic monitoring
Tachycardia or relative bradycardia	Quantitative	Electronic monitoring
Altered mentation	Qualitative	Inspection
